# Modelling of Power-Law Fluid Flow Inside a Piezoelectric Inkjet Printhead

**DOI:** 10.3390/s21072441

**Published:** 2021-04-01

**Authors:** Ju Peng, Jin Huang, Jianjun Wang

**Affiliations:** Key Laboratory of Electronic Equipment Structure Design, Xidian University, Ministry of Education, Xi’an 710071, China; pengju@stu.xidian.edu.cn (J.P.); wangjianjun@xidian.edu.cn (J.W.)

**Keywords:** piezoelectric three-dimensional inkjet printing, power-law fluid, equivalent circuit model, non-Newtonian fluids

## Abstract

Piezoelectric three-dimensional inkjet printing has been used to manufacture heterogeneous objects due to its high level of flexibility. The materials used are non-Newtonian inks with complex rheological properties, and their behavior in the context of inkjet printing has not been fully understood: for example, the fact that the shear-thinning viscosity affects the droplet generation. Therefore, a control strategy coping with shear-thinning behaviors is needed to ensure printing consistency. In this paper, a novel model-based approach is presented to describe the shear-thinning ink dynamics inside the piezoelectric inkjet printhead, which provides the basis to design the excitation parameters in a systematic way. The dynamic equation is simplified into a quasi-one-dimensional equation through the combination of the boundary layer theory and the constitutive equation of the power-law fluid, of which the viscosity is shear-thinning. Based on this, a nonlinear time-varying equivalent circuit model is presented to simulate the power-law fluid flow rate inside the tube. The feasibility and effectiveness of this model can be evaluated by comparing the results of computational fluid dynamics and the experimental results.

## 1. Introduction

Piezoelectric three-dimensional inkjet printing is the combination of droplet injection technology and additive manufacturing technology. With its advantage of high flexibility, it can be used to produce heterogeneous structures and has widened its application to the fields of biology, electronics, and machinery [1,2,3,4]. In most cases, the inks used are suspensions or polymeric liquids, which are rheological complexes and have shear-thinning viscosities [5]. The viscosity has an important influence on droplet generation. The printability of the inks is governed by the Ohnesorge number of the drop, which describes the relative importance of viscous effects to surface tension effects. The Ohnesorge number is defined as follows [6]:

(1) $$\mathrm{Oh}=\frac{\eta }{\sqrt{\rho r\sigma }}$$

where *η* is viscosity, *ρ* is density, *r* is radius, and *σ* is surface tension. For Newtonian fluids, viscosity is independent of flow conditions, and its corresponding Ohnesorge number is viewed as a constant. In the case of shear-thinning fluids, however, viscosity varies with shear rate. Consequently, the excitation printing parameters cannot be determined based on the theory for Newtonian fluids [7,8]. Therefore, a control strategy coping with shear-thinning behaviors is needed to ensure printing consistency.

The excitation parameters, such as the waveform, amplitude, and frequency of the driving voltage, have mostly been sought through experimental trial and error. To investigate waveform effects on droplet generation, Kwon et al. [9] observed the drop formation curve during the printing process, and proposed a method to reduce placement errors caused by satellite drops. Bruce et al. [10] researched the relationship between the characteristics of inks and the jetting rate, the length of tail, and the forming stability of droplets through a series of printing experiments. Using a laser Doppler velocimeter and visualization system to track the droplet generation process, Link et al. [11] analyzed how the amplitude and frequency of the driving voltage influence the inkjet process and droplet formation. By applying double waveforms to the experiment for low-viscosity ink droplet formation, Shin et al. [12] obtained the relationship between the droplet velocity and the amplitude of double waveforms. The constructed control strategies from the above research are empirical, and are based on a significant amount of measured input and output data.

When the parameters of the printhead structure and ink properties are properly identified, computational fluid dynamics (CFD) [13] could be a substitute for experimental methods to analyze the relationships between excitation parameters and inkjet performance. In addition, this method does not require any prior knowledge. In [14], a novel level-set method was presented to describe the interface between air and liquids, and it achieved fine simulation of droplet formation. To deduce the key factors of inkjet performance, Kim et al. [15] combined the simulation of droplet ejection and droplet formation with parametric studies to quantify the printing process. To achieve the stable droplet formation with higher frequency, Aqeel et al. [16] investigated the droplet formation dynamics using a volume-of-fluid method, and obtained the printability range of fluids with different properties. However, the constructed control strategies from the above research consume a great deal of computation, and can only be applied to specific fluids, which makes it difficult to promote them as a viable option.

However, for an efficient control strategy of piezoelectric inkjet printing, a theoretical model that shows the physical mechanism or its combination with the empirical models could be more useful [17]. For one, the method requires less work and avoids waste in experiments. Secondly, the method is a kind of parametric modeling process, which makes the method universal, as long as the parameters of the printhead structure and ink properties are properly identified.

**Figure 1:**
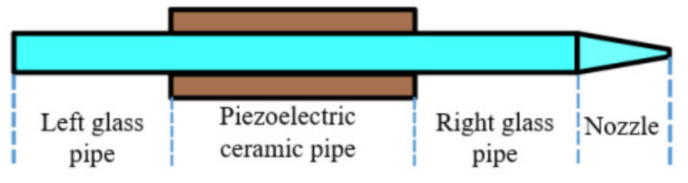
Structure of the squeeze-mode printhead.

The physical mechanism of piezoelectric inkjet printing is derived from the drop-on-demand working principle. In this study, a squeeze-mode piezoelectric inkjet printhead was chosen as the model printhead, and its structure is shown in Figure 1.

Due to the sudden volume change caused by an impulse voltage across the piezoelectric ceramic pipe, pressure waves build up inside the printhead, which start travelling through the inkjet tube. When a positive pressure wave hits the nozzle, the fluid there is pushed outwards. When the amount of kinetic energy transferred outwards is larger than the surface energy needed to form a droplet, a droplet can, in principle, be launched. Whether in reality a droplet is released and what its velocity is depends on the amount of kinetic energy transferred outwards in excess of the surface energy needed to form a droplet. The expected printing performance can be achieved on the condition that the properties of the fluid, the excitation parameters applied on the piezoelectric ceramic tube, and the structure of the channel match with each other.

To understand the mechanism of the piezoelectric inkjet printing, Wijshoff et al. [18] presented a channel acoustics model and obtained the relationship between an excitation signal and a dynamic response of fluid in the frequency domain. With the method of hydrodynamic model analysis, Lee et al. [19] presented an optimization scheme including the frequency of the driving voltage, the properties of the ink, the surrounding temperature and the shape of the interface between the ink and the air. The dynamic lumped element model [20] was proposed to simulate and control the droplet formation process and proved to be simpler than other analytic models. The models in all of the aforementioned studies include a description of the fluid flowing inside the inkjet printhead, which could be the basis for designing the actuation control in a systematic way [21]. However, the models above focused on Newtonian fluids. The ink viscosities were assumed to be constant and the models proposed were not applicable to shear-thinning fluid. Therefore, the control strategies based on them could not ensure the printing precision of the shear-thinning fluid printing process.

In this study, power-law fluid was chosen as the object of study, which has obvious shear-thinning effects. The goal of this paper is to present and contribute a novel model to describe the dynamic response of the power-law fluid inside the inkjet printhead, to predict the flow state at the nozzle. To the best of our knowledge, the idea of the proposed approach has not been considered in previous studies.

This paper is organized as follows: the simplification of the dynamic equation of the power-law fluid inside the inkjet printhead is briefly reviewed in Section 2. The equivalent conversion of the simplified dynamic equation into an equivalent circuit model is explored in detail in Section 3. Section 4 includes an experimental setup and analysis of the completed experiments. Section 5 covers some conclusions and discusses future research.

## 2. Problem Simplification

In this section, the motion equation that describes the flow inside the inkjet printhead is introduced, and the viscous stress term is simplified through the combination of boundary layer theory and the constitution equation of power-law fluids. In the end, the quasi-one-dimensional equation for the power-law fluid flow in the inkjet tube is obtained. As the size of the piezoelectric printhead is small and the displacement of liquid inside the tube is relatively small, the radial displacement of the fluid in the tube is not taken into consideration and the body force and convective acceleration are negligible [20]. Therefore, the Navier-Stokes equation for describing the flow inside the printhead can be simplified as follows:

(2) $$\rho \frac{\partial u}{\partial t}=-\frac{\partial p}{\partial z}+\frac{\partial \tau }{\partial r_{d}}$$

where *u* is the axial velocity of the fluid, *p* is the pressure, and *τ* is the viscous stress while flowing. *z*, *r_d_*, and *t* represent axial direction, radial direction, and time, respectively.

According to the quasi-one-dimensional flow theory in the tube, the velocity of fluid is converted to the flow rate *q* through the integration over the cross section alongside the radial direction. We thus obtain the following equation:

$$\rho \frac{\partial q}{\partial t}=-\pi r_{0}{}^{2}\frac{\partial p}{\partial z}+2\pi r_{0}\tau -2\pi \int_{0}^{r_{0}}\tau dr_{d}$$

where *r_0_* is the radius of the cylindrical tube, $2\pi r_{0}\tau$ is the shear stress on the wall, and $2\pi \int_{0}^{r_{0}}\tau dr_{d}$ represents the integration of viscous stress in the main flow.

The ratio of the change in radial direction to the radius is so small that the flow inside the tube could be assumed to be an undeveloped flow. It is in a tiny boundary layer that the viscous stress affects the liquid in the channel, and there is rarely viscous stress in the main flow [22]. Therefore, when the inkjet printhead is operating, the viscous stress inside can be approximated as the shearing stress on the wall of the inkjet tube, and $2\pi \int_{0}^{r_{0}}\tau dr_{d}$ can be omitted. Assuming this to be true, we can obtain the following quasi-one-dimensional equation:

(3) $$\rho \frac{\partial q}{\partial t}=-\pi r_{0}{}^{2}\frac{\partial p}{\partial z}+2\pi r_{0}\tau$$

In order to describe how the shear-thinning behaviors of non-Newtonian fluids influence the flow state inside the printhead, power-law fluid was chosen as the object of the study, of which the viscosity varies with the fluid motion [23]. The constitutive equation of power-law fluid describes the relationship between the viscous stress and the velocity gradient, which is shown as:

(4) $$\tau =\mu {\left(\dot{\gamma }\right)}^{n}$$

where $\dot{\gamma }=\partial u/\partial r$ denotes the velocity gradient along the radial direction, *μ* denotes the viscosity factor, and *n* denotes the power-law factor. The constitutive equation can also be presented in a Newton-like form:

(5) $$\tau =\eta_{eff}\left(\dot{\gamma }\right)\cdot \dot{\gamma },\;\eta_{eff}\left(\dot{\gamma }\right)=\mu {\left(\dot{\gamma }\right)}^{n-1}$$

where $\eta_{eff}\left(\dot{\gamma }\right)$ stands for the Newton-like viscosity.

In the inkjet tube, the radial displacement happens in the radial direction of the piezoelectric ceramic pipe when excitation voltage works, and the resulting pressure causes the fluid inside to flow. Therefore, the motion of fluid inside the tube can be viewed as an oscillating flow [24]. Since the oscillating flow inside the inkjet tube could be viewed as a Stokes boundary flow [25], assuming that the density of the fluid is constant during the oscillatory flow, the oscillating frequency is expressed as [26]:

(6) $$\omega =\frac{2\pi }{T}=\frac{2\pi }{2l_{T}/c}=\frac{\pi c}{l_{T}}$$

where *l_T_* is the total length of the printhead and *c* is the acoustic velocity of the fluid.

The velocity of the main flow *u_m_*, the velocity of the boundary layer *u_v_*, and the thickness of the boundary layer *δ,* meet the relationship [25]:

(7) $$\frac{u_{m}-u_{v}}{u_{m}}=e^{-k\delta },k=\sqrt{\frac{\omega \rho }{2\eta_{eff}}}=\sqrt{\frac{\pi \rho c}{2\eta_{eff}l_{T}}}$$

In [22], the description of the thickness of the viscous boundary layer *δ_v_* is defined as:

(8) $$\delta_{v}=\frac{1}{k}=\sqrt{\frac{2\eta_{eff}l_{T}}{\pi \rho c}}$$

As mentioned above, compared with the thickness of the main flow, the boundary layer is so thin that the velocity gradient inside can be assumed to be linear, and simplified as:

(9) $$\dot{\gamma }=\frac{u_{m}}{\delta_{v}}=u_{m}\sqrt{\frac{\pi \rho c}{2\eta_{eff}l_{T}}}$$

By plugging (9) into (8), the expression of the thickness of the boundary layer *δ_v_* can be written as:

(10) $$\delta_{v}=\sqrt[{n+1}]{2\mu u_{m}{}^{n-1}l_{T}/\left(\pi \rho c\right)}$$

By plugging (10) into the constitutive equation of power-law fluid, the equivalent form of the viscous stress of the power-law fluid is derived as:

(11) $$\tau =\mu^{\frac{1}{n+1}}u_{m}{}^{\frac{2n}{n+1}}{\left(\pi \rho c/2l_{T}\right)}^{\frac{n}{n+1}}$$

The velocity of fluid *u_m_* can be converted to the flow rate *q*, assuming that the integration of the main flow over the cross section approximates the flow rate. By plugging (11) into (3), the quasi-one-dimensional equation is converted into a pressure drop equation that means the pressure change of the two ends of the tube consists of the inertia force pressure drop and viscous stress pressure drop through the transposition, which is shown as:

(12) $$\frac{\partial p}{\partial z}=-\frac{\rho }{\pi r_{0}{}^{2}}\frac{\partial q}{\partial t}+2\pi^{\frac{-n}{n+1}}r_{0}{}^{\frac{-1-5n}{n+1}}\mu^{\frac{1}{n+1}}q^{\frac{2n}{n+1}}{\left(\rho c/2l_{T}\right)}^{\frac{n}{n+1}}$$

## 3. Equivalent Conversion

According to the similarity between fluid flow and current flow [27], an equivalent circuit model is presented to reveal the physical mechanism of the fluid flow inside the inkjet tube.

In the model, the voltage *U*, voltage change ΔU, and current *I* in the circuit are equivalent to the pressure *p*, pressure change Δp, and volume flow rate *q* in the fluid, respectively. Due to the ability to impede the change of current, the inductor *L* is equivalent to the inertia of the fluid. The resistor *R* is equivalent to the viscous drag of the fluid because of the ability to impede the current flow. Therefore, (12) can be converted to the equivalent circuit expression:

(13) $$\Delta U=-L\frac{\partial I}{\partial t}+RI$$

**Figure 2:**
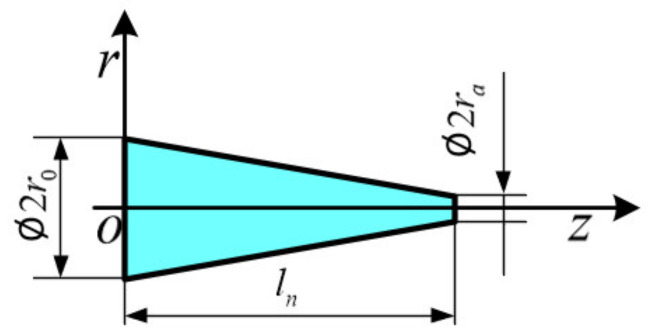
The structure parameters of the conical tube.

As shown in Figure 1, the piezoelectric inkjet printhead consists of two kinds of tubes: a pipe and a nozzle. For the pipe, through the integration along the tube’s axial direction, the corresponding equivalent form is:

(14) $$\Delta p=-\frac{\rho l}{\pi r_{0}{}^{2}}\frac{\partial q}{\partial t}+2l\pi^{\frac{-n}{n+1}}r_{0}{}^{\frac{-1-5n}{n+1}}\mu^{\frac{1}{n+1}}q^{\frac{2n}{n+1}}{\left(\rho /2l_{T}\right)}^{\frac{n}{n+1}}$$

where *l* is the length of the cylindrical tube. For the conical tube, the radius of the cross section varies along the axial direction, as shown in Figure 2.

The radius of the conical tube can be expressed as:

(15) $$r\left(z\right)=r_{0}+\frac{r_{a}-r_{0}}{l_{n}}z$$

where *l_n_* is the length of the conical tube and *r_a_* is the radius of the nozzle outlet.

By plugging (15) into (12), we obtain the transformation shown as:

(16) $$\frac{\partial p}{\partial z}=-\frac{\rho }{\pi r{\left(z\right)}^{2}}\frac{\partial q}{\partial t}+2\pi^{\frac{-n}{n+1}}r{\left(z\right)}^{\frac{-1-5n}{n+1}}\mu^{\frac{1}{n+1}}q^{\frac{2n}{n+1}}{\left(\rho c/2l_{T}\right)}^{\frac{n}{n+1}}$$

By integrating (16) along the tube’s axial direction, the corresponding equivalent form is shown as:

(17) $$\Delta p=-\frac{\rho l_{n}}{\pi r_{a}r_{0}}\frac{\partial q}{\partial t}+\pi^{\frac{-n}{n+1}}\mu^{\frac{1}{n+1}}q^{\frac{2n}{n+1}}{\left(\rho c/2l_{T}\right)}^{\frac{n}{n+1}}\frac{n+1}{2n}\frac{l_{n}}{r_{0}-r_{a}}\left[r_{a}{}^{-\frac{4n}{n+1}}-r_{0}{}^{-\frac{4n}{n+1}}\right]$$

From (13), (14), and (17), it can be seen that all the resistors are current-varying. For the pipe, the corresponding resistor is:

$$R=2l\pi^{\frac{-n}{n+1}}r_{0}{}^{\frac{-1-5n}{n+1}}\mu^{\frac{1}{n+1}}I^{\frac{n-1}{n+1}}{\left(\rho /2l_{T}\right)}^{\frac{n}{n+1}}$$

For the conical tube, the corresponding resistor is:

$$R_{n}=\pi^{\frac{-n}{n+1}}\mu^{\frac{1}{n+1}}I^{\frac{2n}{n+1}}{\left(\rho c/2l_{T}\right)}^{\frac{n}{n+1}}\frac{n+1}{2n}\frac{l_{n}}{r_{0}-r_{a}}\left[r_{a}{}^{-\frac{4n}{n+1}}-r_{0}{}^{-\frac{4n}{n+1}}\right]$$

At the initial moment, the fluid inside the printhead is assumed to be a Newtonian fluid, as it does not show shear thinning. Therefore, for the pipe, the initial value *R_i_* of the corresponding resistor is:

$$R_{i}=lr_{0}{}^{-3}\sqrt{2\mu \rho c/\pi l_{T}}$$

For the pipe, the initial value *R_in_* of the corresponding resistor is:

$$R_{in}=l_{n}\sqrt{\mu \rho c/2\pi l_{T}}\frac{r_{0}+r_{a}}{r_{0}{}^{2}r_{a}{}^{2}}$$

As a component that can hold a small amount of electrical charge, the capacitor *C* is equivalent to the fluid compressibility, the fluid surface tension effect, and the tiny volume change of the tube.

The evaluations of the capacitors in the relevant equivalent circuit model need the derivation of the corresponding equation of mass conservation during the ink-jetting process, which is shown as:

(18) $$\frac{D\rho }{Dt}+\rho \left(\nabla \cdot \vec{u}\right)=0$$

where Dρ/Dt is the material derivative, which means how the density of the fluid element varies with time and position. As the flow inside the tube is oscillatory, the material derivative can be simplified to ∂ρ/∂t [28]. $\rho \left(\nabla \cdot \vec{u}\right)$ is the mass change caused by the inflow and outflow of fluid inside the fluid element. As only the axial motion is taken into consideration, this term is reduced to $\rho_{0}\partial u/\partial z$, where *ρ_0_* represents the initial density of the fluid. Therefore, (18) can be simplified as:

(19) $$\frac{\partial \rho }{\partial t}=-\rho_{0}\frac{\partial u}{\partial z}$$

By integrating (19) over the cross section, axial direction, and time, the resulting expression is:

(20) $$d\rho =-\frac{\rho_{0}}{V}\int \Delta qdt$$

where *V* is the volume of the tube. Furthermore, the density in the tube can be expressed as:

(21) $$\rho =\rho_{0}-\frac{\rho_{0}}{V}\int \Delta qdt$$

The transmission of pressure waves inside the tube is transient enough to be assumed to be adiabatic. The liquid inside obeys the state equation $dp=c^{2}d\rho$ [20]. Thus, (21) can be written as:

(22) $$p=c^{2}\rho_{0}-\frac{c^{2}\rho_{0}}{V}\int \Delta qdt$$

where *c*^2^*ρ_0_* is the initial pressure inside the tube. By converting the equation into the equivalent circuit form, which means the voltage applied to both ends of the capacitor, it can be expressed as:

(23) $$U=U_{pp}-\frac{dQ}{C},\int \Delta qdt=\Delta Q,U_{pp}=c^{2}\rho_{0}$$

where *Q* is the quantity of electric charge and *U_pp_* is the initial voltage of the circuit. Therefore, it is easy to obtain the expression of the capacitor that represents the fluid compressibility in the tube from (23). For the pipe, the corresponding capacitor *C_l_* form is:

$$C_{l}=\frac{l\pi r_{0}^{2}}{c^{2}\rho_{0}}$$

For the conical tube, the corresponding capacitor *C_n_* form is:

$$C_{n}=\frac{l_{n}\pi \left(r_{0}^{2}+r_{0}r_{a}+r_{a}^{2}\right)}{3c^{2}\rho_{0}}$$

When the piezoelectric printhead operates, the radius *r* of the piezoelectric ceramic pipe varies with time, which can be expressed in the following form:

(24) $$r\left(t\right)=r_{0}-V\left(t\right)d$$

where *V*(*t*) is the actuation voltage and *d* is the conversion coefficient of the inverse piezoelectric effects [29]. The area of the pipe’s cross section is expressed as *πr*(*t*)^2^, and the form of the time-varying capacitor *C_p_* that represents the volume change of the tube is:

$$C_{p}=\frac{l_{p}\pi \left[V{\left(t\right)}^{2}d^{2}-2r_{0}V\left(t\right)d\right]}{c^{2}\rho_{0}}$$

where *l_p_* is the length of the piezoelectric ceramic pipe.

Capacitor *C_s_* is equivalent to the fluid surface tension effect, which can cause pressure change at the outlet of the nozzle. The average values of the flow rate at the nozzle outlet are roof-shaped to estimate the expression of the capacitor parameter [30], which is shown as:

(25) $$C_{s}=\frac{Q}{U}=\frac{V_{men}}{p_{men}}=\frac{\pi r_{a}^{4}}{3\sigma }$$

where *V_men_* is the roof-shaped volume of the fluid and *p_men_* is the pressure change at the nozzle outlet.

**Figure 3:**
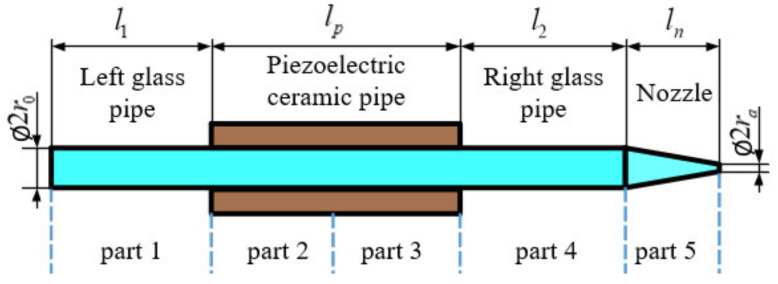
The division of the inkjet printhead and structure parameters.

Because it is easy to observe the fluid compressibility at the junctions between the pipes and the pressure waves that propagate from the middle of the piezoelectric ceramic pipe to both ends of the inkjet channel, the squeeze-mode piezoelectric inkjet printhead is divided into five parts, one of which (the piezoelectric ceramic pipe) is divided into two equal parts. The division and structure parameters are shown in Figure 3.

The piezoelectric inkjet printhead is equivalent to a closed circuit and every part can be reviewed as a circuit branch, of which the pressure drop could be described by (14) and (17). Between part 1 and part 2, part 3 and part 4, and part 4 and part 5, fluid compressibility plays the leading role. At the junction between part 2 and part 3, the volume change of the tube takes effect. At the outlet of the nozzle, surface tension operates. At the two ends of the inkjet channel, the surrounding pressure should be taken into consideration.

**Figure 4:**
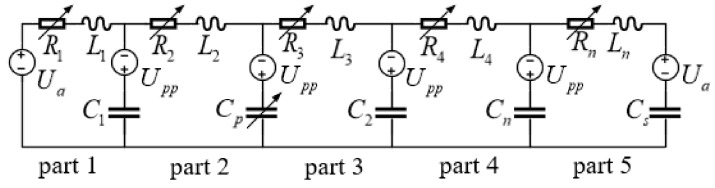
Equivalent circuit diagram.

From the above analysis, the corresponding equivalent circuit diagram of the inkjet printhead is shown in Figure 4, where *Ua* represents the surrounding pressure with the value of 1 atm.

The corresponding equivalent circuit parameters are listed in Table 1.

Based on the above circuit diagram and the parameter table, several equations can be derived according to Kirchhoff’s voltage law and Kirchhoff’s current law to represent the equivalent circuit model:

(26) $$\left\{\begin{array}{cc} \; & \frac{du_{c1}\left(t\right)}{dt}=\frac{1}{C_{1}}i_{1}\left(t\right)-\frac{1}{C_{1}}i_{2}\left(t\right)\\ \; & \frac{du_{c2}\left(t\right)}{dt}=-\frac{1}{C_{p}\left(t\right)}\frac{dC_{p}\left(t\right)}{dt}u_{2}\left(t\right)+\frac{1}{C_{p}\left(t\right)}i_{2}\left(t\right)-\frac{1}{C_{p}\left(t\right)}i_{3}\left(t\right)\\ \; & \frac{du_{c3}\left(t\right)}{dt}=\frac{1}{C_{2}}i_{3}\left(t\right)-\frac{1}{C_{2}}i_{4}\left(t\right)\\ \; & \frac{du_{c4}\left(t\right)}{dt}=\frac{1}{C_{n}}i_{4}\left(t\right)-\frac{1}{C_{n}}i_{5}\left(t\right)\\ \; & \frac{du_{c5}\left(t\right)}{dt}=\frac{1}{C_{s}}i_{5}\left(t\right)\\ \; & \frac{di_{1}\left(t\right)}{dt}=-\frac{1}{L_{1}}u_{c1}\left(t\right)-\frac{R_{1}\left(i_{1}\left(t\right)\right)}{L_{1}}i_{1}\left(t\right)+\frac{1}{L_{1}}\left(U_{a}+Upp\right)\\ \; & \frac{di_{2}\left(t\right)}{dt}=\frac{1}{L_{2}}u_{c1}\left(t\right)-\frac{1}{L_{2}}u_{c2}\left(t\right)-\frac{R_{2}\left(i_{2}\left(t\right)\right)}{L_{2}}i_{2}\left(t\right)\\ \; & \frac{di_{3}\left(t\right)}{dt}=\frac{1}{L_{3}}u_{c2}\left(t\right)-\frac{1}{L_{3}}u_{c3}\left(t\right)-\frac{R_{3}\left(i_{3}\left(t\right)\right)}{L_{3}}i_{3}\left(t\right)\\ \; & \frac{di_{4}\left(t\right)}{dt}=\frac{1}{L_{4}}u_{c3}\left(t\right)-\frac{1}{L_{4}}u_{c4}\left(t\right)-\frac{R_{4}\left(i_{4}\left(t\right)\right)}{L_{4}}i_{4}\left(t\right)\\ \; & \frac{di_{5}\left(t\right)}{dt}=\frac{1}{L_{n}}u_{c4}\left(t\right)-\frac{1}{L_{n}}u_{c5}\left(t\right)-\frac{R_{n}\left(i_{5}\left(t\right)\right)}{L_{n}}i_{5}\left(t\right)-\frac{1}{L_{n}}\left(U_{a}+Upp\right) \end{array}\right.$$

In the above equations, *u_c_*_1_(*t*), *u_c_*_2_(*t*), *u_c_*_3_(*t*), *u_c_*_4_(*t*), and *u_c_*_5_(*t*) represent the terminal voltage of the capacitors *C*_1_, *C_p_*, *C*_2_, *C_n_,* and *C_s_* respectively, which implies the absolute pressure at the junctions between the parts. *R*_1_(*i*_1_(*t*)), *R*_2_(*i*_2_(*t*)), *R*_3_(*i*_3_(*t*)), *R*_4_(*i*_4_(*t*)), and *R*_n_(*i*_5_(*t*)) are current-varying resistors while *i*_1_(*t*), *i*_2_(*t*), *i*_3_(*t*), *i*_4_(*t*), and *i*_5_(*t*) represent the currents through the corresponding resistors, which means the flow rates in the corresponding parts. By choosing the *u_c_*_1_(*t*), *u_c_*_2_(*t*), *u_c_*_3_(*t*), *u_c_*_4_(*t*) *u_c_*_5_(*t*), *i*_1_(*t*), *i*_2_(*t*), *i*_3_(*t*), *i*_4_(*t*), and *i*_5_(*t*) as state variables, the above equations can be converted to the state equation of a nonlinear time-varying system:

(27) $$\dot{y}\left(t\right)=M(t)y(t)+Nw$$

In the above equation,

$$\dot{y}\left(t\right)=\left[\begin{array}{cccccccccc} \frac{du_{c1}(t)}{dt} & \frac{du_{c2}(t)}{dt} & \frac{du_{c3}(t)}{dt} & \frac{du_{c4}(t)}{dt} & \frac{du_{c5}(t)}{dt} & \frac{di_{1}(t)}{dt} & \frac{di_{2}(t)}{dt} & \frac{di_{3}(t)}{dt} & \frac{di_{4}(t)}{dt} & \frac{di_{5}(t)}{dt} \end{array}\right]\;y\left(t\right)={\left[\begin{array}{cccccccccc} u_{c1}(t) & u_{c2}(t) & u_{c3}(t) & u_{c4}(t) & u_{c5}(t) & i_{1}(t) & i_{2}(t) & i_{3}(t) & i_{4}(t) & i_{5}(t) \end{array}\right]}^{T}\;N=\left[\begin{array}{cccccccccc} 0 & 0 & 0 & 0 & 0 & \frac{1}{L_{1}} & 0 & 0 & 0 & -\frac{1}{L_{n}} \end{array}\right]\;\;\;w=\left[U_{a}+U_{pp}\right]\;\begin{array}{c} M\left(t\right)=\left[\begin{array}{cccccccccc} 0 & 0 & 0 & 0 & 0 & \frac{1}{C_{1}} & -\frac{1}{C_{1}} & 0 & 0 & 0\\ -\frac{1}{C_{p}\left(t\right)}\frac{dC_{p}\left(t\right)}{dt} & 0 & 0 & 0 & 0 & 0 & \frac{1}{C_{p}\left(t\right)} & -\frac{1}{C_{p}\left(t\right)} & 0 & 0\\ 0 & 0 & 0 & 0 & 0 & 0 & 0 & \frac{1}{C_{2}} & -\frac{1}{C_{2}} & 0\\ 0 & 0 & 0 & 0 & 0 & 0 & 0 & 0 & \frac{1}{C_{n}} & -\frac{1}{C_{n}}\\ 0 & 0 & 0 & 0 & 0 & 0 & 0 & 0 & 0 & \frac{1}{C_{s}}\\ -\frac{1}{L_{1}} & 0 & 0 & 0 & 0 & -\frac{R_{1}\left(i_{1}\left(t\right)\right)}{L_{1}} & 0 & 0 & 0 & 0\\ \frac{1}{L_{2}} & -\frac{1}{L_{2}} & 0 & 0 & 0 & 0 & -\frac{R_{2}\left(i_{2}\left(t\right)\right)}{L_{2}} & 0 & 0 & 0\\ 0 & \frac{1}{L_{3}} & -\frac{1}{L_{3}} & 0 & 0 & 0 & 0 & -\frac{R_{3}\left(i_{3}\left(t\right)\right)}{L_{3}} & 0 & 0\\ 0 & 0 & \frac{1}{L_{4}} & -\frac{1}{L_{4}} & 0 & 0 & 0 & 0 & -\frac{R_{4}\left(i_{4}\left(t\right)\right)}{L_{4}} & 0\\ 0 & 0 & 0 & \frac{1}{L_{n}} & -\frac{1}{L_{n}} & 0 & 0 & 0 & 0 & -\frac{R_{n}\left(i_{5}\left(t\right)\right)}{L_{n}} \end{array}\right] \end{array}$$

The pressure at the junction between parts and the flow rates in every part can be determined by solving (27) with the recursive solution method mentioned in [26].

## 4. Results and Discussion

There has been much research carried out about the experimental detections and two-phase flow simulations of the drop formation at the nozzle.

As the fluid flow inside the printhead cannot be detected directly, it is hard to verify the accuracy of the proposed model through a comparison with the experimental results. However, the results of the proposed model could be used as the boundary conditions to simulate the drop formation at the nozzle by CFD. Then, a comparison between the results of CFD and the experimental results could be made to evaluate the feasibility and effectiveness of the proposed model.

To verify the accuracy of the proposed model, shear-thinning fluids were prepared by dissolving polyacrylamide (PAM) in deionized (DI) water, which has been reported to be a power-law fluid [31].

The rheological properties of the liquids were measured using a visualization rheometer (MCR302, Anton-Paar, Graz, Austria). The surface tension was measured using the Du Nouy ring method (DCAT25 Tensiometer, Dataphysics Instruments, Stuttgart, Germany).

**Figure 5:**
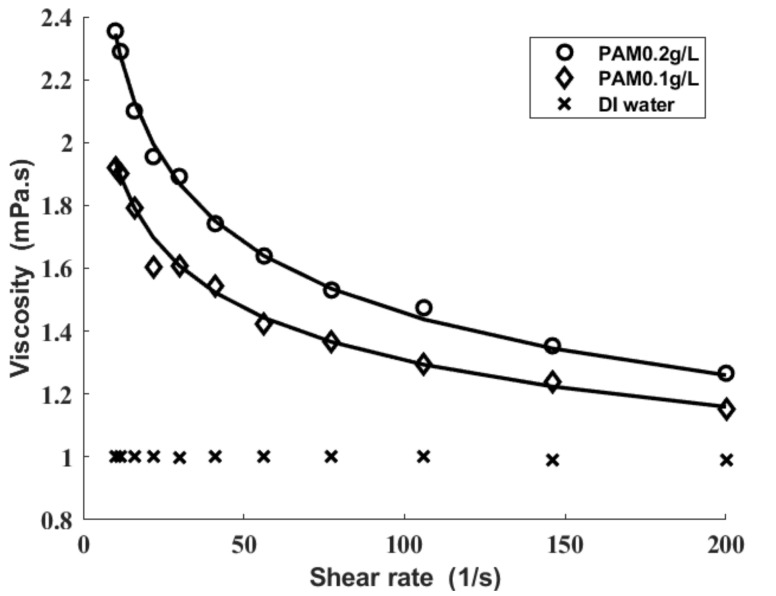
Viscosities of polyacrylamide (PAM) solutions for differing PAM concentrations. The symbols are measured values. The solid lines are the power-law model fit.

Figure 5 shows the shear viscosities of the fluids tested in the present research. The DI water has shear-independent viscosities while both of the PAM solutions have shear-thinning viscosities. The viscosity of the polyacrylamide solution is fitted to the power-law model [32].

$$\eta =\mu {\left(\dot{\gamma }\right)}^{n-1}$$

The corresponding rheological parameters are listed in Table 2.

As the PAM concentrations were so small that the density of the PAM solutions tested in the present research was similar to DI water, acoustic speed in DI water was used as the acoustic speed in PAM solutions. As a result, c=1482m/s and $\rho =1000\mathrm{kg}/m^{3}$.

The droplet formation detection experimental system was established in [23]. An MJ-AL-80 piezoelectric printhead (MicroFab, Plano, TX, USA) was used to eject droplets, which is a kind of squeeze-mode piezoelectric printhead. The structure parameters of the printhead were determined through measurements, which are listed in Table 3.

The relevant conversion coefficient d of inverse piezoelectric effects approximates $0.32\times 10^{-9}\left(m/v\right)$.

**Figure 6:**
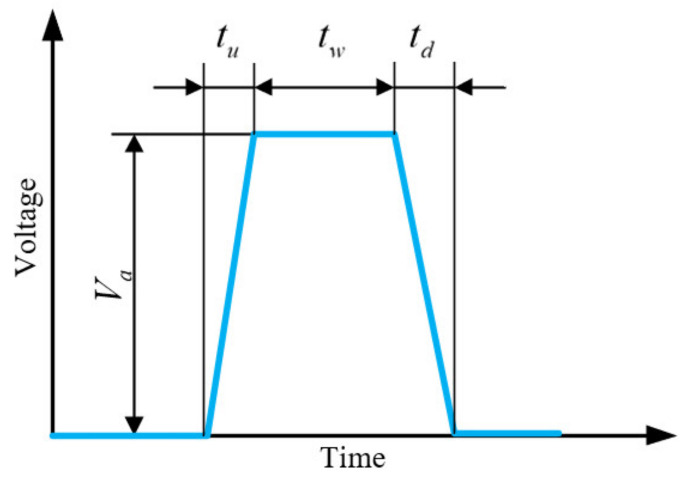
Standard single trapezoidal pulse waveform.

The standard single trapezoidal pulse waveform [22] was applied to generate the droplet, which is shown in Figure 6.

In the experiment, the actuation parameters were determined by trial and error. Through the adjustments, the stable inkjet printing processes were obtained and the corresponding actuation parameters are shown in Table 4.

**Figure 7:**
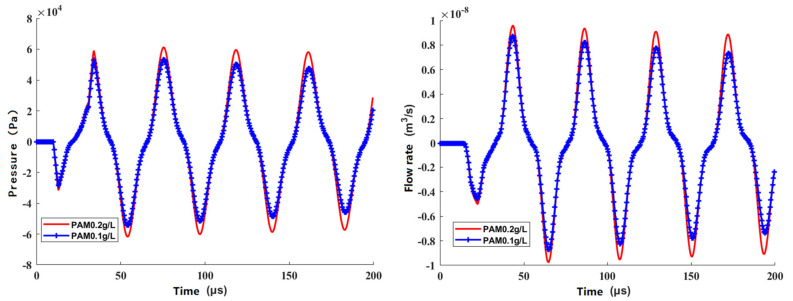
Results of the proposed model.

We took the rheological properties, the structure parameters, and actuation parameters into the equivalent circuit model. The pressure and velocity at the nozzle were obtained through a recursive solution method [29]. Figure 7 shows that the relationship of pressure to velocity varies with time.

**Figure 8:**
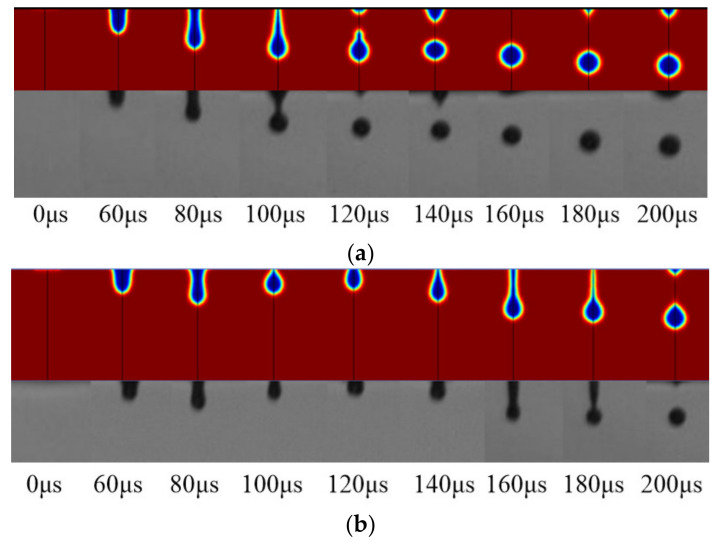
(**a**) Comparison of power-law fluid (PAM 0.2g/L) computational fluid dynamics (CFD) results and experimental results; (**b**) Comparison of power-law fluid (PAM 0.1g/L) CFD results and experimental results.

We then set the results as the boundary conditions of the CFD two-phase fluid model to simulate the drop formation at the nozzle. The experimental results and simulation results were consistent in time, which is shown in Figure 8 and proves the feasibility and effectiveness of the model.

## 5. Conclusions

In this research, we proposed an equivalent circuit model to describe the dynamic response of power-law fluid flow inside a squeeze-mode piezoelectric printhead. The nonlinear viscous stress term was simplified through the combination of boundary layer theory and the constitutive equation of power-law fluids. The corresponding equivalent parameters were derived according to the physical significance. The relationship between pressure and velocity varied with time at the nozzle outlet, which was obtained from the proposed model, and were set as the boundary conditions of the CFD two-phase fluid model, which simulates the droplet formation of polyacrylamide solutions in DI water in inkjet printing. The results of droplet simulation were consistent in time with the experimental results, which proves the feasibility and effectiveness of the proposed model. This model reveals the physical mechanism of power-law fluid inkjet printing, and thus, the proposed model opens the door for a specific way of designing the excitation parameters systematically.

## Figures and Tables

**Table 1:** Equivalent circuit parameters.

Equivalent Circuit Parameters	Expressions
$R_{1}$	$2l_{1}\pi^{\frac{-n}{n+1}}r_{0}{}^{\frac{-1-5n}{n+1}}\mu^{\frac{1}{n+1}}{\left(\rho c/2l_{T}\right)}^{\frac{n}{n+1}}I^{\frac{n-1}{n+1}}$
$R_{2}$	$l_{p}\pi^{\frac{-n}{n+1}}r_{0}{}^{\frac{-1-5n}{n+1}}\mu^{\frac{1}{n+1}}{\left(\rho c/2l_{T}\right)}^{\frac{n}{n+1}}I^{\frac{n-1}{n+1}}$
$R_{3}$	$l_{p}\pi^{\frac{-n}{n+1}}r_{0}{}^{\frac{-1-5n}{n+1}}\mu^{\frac{1}{n+1}}{\left(\rho c/2l_{T}\right)}^{\frac{n}{n+1}}I^{\frac{n-1}{n+1}}$
$R_{4}$	$2l_{2}\pi^{\frac{-n}{n+1}}r_{0}{}^{\frac{-1-5n}{n+1}}\mu^{\frac{1}{n+1}}{\left(\rho c/2l_{T}\right)}^{\frac{n}{n+1}}I^{\frac{n-1}{n+1}}$
$R_{n}$	$\frac{n+1}{2n}\pi^{\frac{-n}{n+1}}\mu^{\frac{1}{n+1}}{\left(\rho c/2l_{T}\right)}^{\frac{n}{n+1}}\frac{l_{n}}{r_{0}-r_{a}}\left[r_{a}{}^{-\frac{4n}{n+1}}-r_{0}{}^{-\frac{4n}{n+1}}\right]I^{\frac{n-1}{n+1}}$
$R_{i1}$	$l_{1}r_{0}{}^{-3}\sqrt{2\mu \rho c/\pi l_{T}}$
$R_{i2}$	$l_{2}r_{0}{}^{-3}\sqrt{\mu \rho c/2\pi l_{T}}$
$R_{i3}$	$l_{2}r_{0}{}^{-3}\sqrt{\mu \rho c/2\pi l_{T}}$
$R_{i4}$	$l_{3}r_{0}{}^{-3}\sqrt{2\mu \rho c/\pi l_{T}}$
$R_{in}$	$l_{n}\sqrt{\mu \rho c/2\pi l_{T}}\frac{r_{0}+r_{a}}{r_{0}{}^{2}r_{a}{}^{2}}$
$C_{1}$	$\left(l_{1}+\frac{l_{p}}{2}\right)\frac{\pi r_{0}{}^{2}}{c^{2}\rho_{0}}$
$C_{p}$	$\frac{l_{p}\pi \left[V{\left(t\right)}^{2}d^{2}-2r_{0}V\left(t\right)d\right]}{c^{2}\rho_{0}}$
$C_{2}$	$\left(l_{2}+\frac{l_{p}}{2}\right)\frac{\pi r_{0}{}^{2}}{c^{2}\rho_{0}}$
$C_{n}$	$\frac{l_{n}\pi \left(r_{0}^{2}+r_{0}r_{a}+r_{a}^{2}\right)}{3c^{2}\rho_{0}}$
$C_{s}$	$\frac{\pi r_{a}^{4}}{3\sigma }$
$L_{1}$	$\frac{\rho l_{1}}{\pi r_{0}{}^{2}}$
$L_{2}$	$\frac{\rho l_{p}}{2\pi r_{0}{}^{2}}$
$L_{3}$	$\frac{\rho l_{p}}{2\pi r^{2}}$
$L_{4}$	$\frac{\rho l_{2}}{\pi r^{2}}$
$L_{n}$	$\frac{\rho l_{n}}{\pi r_{a}r_{0}}$
$U_{a}$	$1atm$
$U_{pp}$	$c^{2}\rho_{0}$

**Table 2:** Rheological parameters.

Solutions	Viscosity Factor *μ*	Power-Law Factor *n*	Surface Tension *σ* (mN/m)
PAM 0.2g/L	3.783	0.7925	66.8
PAM 0.1g/L	2.887	0.8278	64.5

**Table 3:** Structure parameters of printhead.

**Length (mm)**	$l_{1}$	$l_{2}$	$l_{3}$	$l_{4}$	$r_{0}$	$r_{a}$
	8.87	8.2	4.71	1	0.235	0.04

**Table 4:** Actuation parameters.

	$V_{a}\left(v\right)$	$t_{u}\left(\mu s\right)$	$t_{w}\left(\mu s\right)$	$t_{d}\left(\mu s\right)$
PAM 0.2g/L	37	3	18	3
PAM 0.1g/L	32	3	18	3
